# Identification of Druggable Targets for Alzheimer's Disease by Analyzing Circulating Inflammatory Proteins With Mendelian Randomization

**DOI:** 10.1002/brb3.70797

**Published:** 2025-08-27

**Authors:** Hongliang An, Jianhong Gu, Taiping Li

**Affiliations:** ^1^ Department of Pharmacy Nanjing Meishan Hospital Nanjing China; ^2^ Child Mental Health Research Center Nanjing Brain Hospital Affiliated to Nanjing Medical University Nanjing China; ^3^ Neuro‐Psychiatric Institute Nanjing Brain Hospital Affiliated to Nanjing Medical University Nanjing China

**Keywords:** Alzheimer's disease, circulating inflammatory proteins, druggable targets, Mendelian randomization

## Abstract

**Objective:**

To identify potential druggable targets for Alzheimer's disease (AD) by analyzing circulating inflammatory proteins using Mendelian randomization (MR).

**Methods:**

Two‐sample MR analysis was employed to investigate the causal relationships between 91 circulating inflammatory proteins and AD. The primary MR method utilized was the inverse variance weighted (IVW) model, while the weighted median (WM) and MR‐Egger models were applied for sensitivity analysis. To assess the heterogeneity of instrumental variables (IVs), Cochran's *Q*‐test and *I* ^2^ statistics were utilized. Additionally, ChEMBL and DGIdb databases with Bayesian colocalization analysis were consulted to identify potential druggable proteins.

**Results:**

MR analysis identified eight inflammatory proteins significantly associated with AD risk. Among these proteins, TNFB [odds ratio (OR): 1.06, 95% Confidence Interval (CI): 1.02–1.11, *p* = 8.77×10^−^^3^], TSLP (OR: 1.10, 95% CI: 1.01–1.19, *p* = 0.028), S100A12 (OR: 1.09, 95% CI: 1.01–1.18, *p* = 0.03), CD244 (OR: 1.07, 95% CI: 1.00–1.13, *p* = 0.036), and IL33 (OR: 1.08, 95% CI: 1.00 –1.17, *p* = 0.048) were identified as proteins associated with elevated AD risk. Conversely, three inflammatory proteins exhibited a protective effect against AD, including NRTN (OR: 0.91, 95% CI: 0.85–0.99; *p* = 0.019), CCL4 (OR: 0.95, 95% CI: 0.91–1.00, *p* = 0.029), and MMP1 (OR: 0.93, 95% CI: 0.87–1.00, *p* = 0.049). Notably, according to the gene‐drug analysis, TSLP, S100A12, CD244, CCL4, and MMP1 were identified as druggable. Additionally, MMP1 (PP4 = 0.92) and CCL4 (PP4 = 0.87) in the prefrontal cortex had the strongest colocalization evidence (PP4 > 0.85), suggesting they could potentially serve as novel therapeutic targets for AD.

**Conclusions:**

Integrative genetic analyses indicate that genetically determined circulating levels of TSLP, S100A12, CD244, CCL4, and MMP1 exert causal effects on AD risk. These findings nominate all five proteins as potential therapeutic targets, with MMP1 and CCL4 representing priority candidates warranting further mechanistic investigation and clinical validation.

## Abstract 2

Mendelian Randomization (MR) identifies causal links between inflammatory proteins (e.g., TSLP, S100A12) and Alzheimer's disease (AD) risk, revealing druggable targets for therapeutic development. Protective proteins (e.g., CCL4, MMP1) offer additional avenues for AD intervention.

**Abbreviations**
AD: Alzheimer's disease
Aβ: Amyloid beta
CCL4: C‐C motif chemokine 4
CD244: Natural killer cell receptor 2B4
CI: Confidence Interval
eQTL: expression quantitative trait loci
GWAS: Genome‐Wide Association Study
IGAP: International Genomics of Alzheimer's Project
IL‐1β: Interleukin‐1β
IL33: Interleukin‐33
IVs: instrumental variables
IVW: inverse variance weighted
MCI: Mild cognitive impairment
MMP1: Matrix metalloproteinase‐1
MMPs: Matrix metalloproteinases
MR: Mendelian Randomization
NRTN: Neurturin
OR: Odds Ratio
PPARγ: Peroxisome proliferator‐activated receptor γ
pQTLs: protein quantitative trait loci
rTMS: repetitive transcranial magnetic stimulation
S100A12: Protein S100‐A12
SNPs: Single nucleotide polymorphisms
TNFB: Tumor necrosis factor β
TSLP: Thymic stromal lymphopoietin
VCAM‐1: Vascular cell adhesion molecule‐1
WM: weighted median

## 1 Introduction

The aging of the global population is a significant factor contributing to the rising incidence of Alzheimer's disease (AD), which stands as the foremost cause of dementia worldwide (Jason and Andrew 2018; Zhao et al. 2024). This demographic shift, characterized by an increasing proportion of elderly individuals, has profound implications for public health and healthcare systems. AD confers a heavy burden on the society by causing excess morbidity and mortality (Lanctôt et al. 2024; Agudelo‐Botero et al. 2023). Key pathological hallmarks of AD include the deposition of amyloid beta (Aβ) and hyperphosphorylated tau protein, as well as neuroinflammation mediated by glial cells (Kiara et al. 2022). These pathological processes are intricately linked and contribute to the progressive cognitive decline and memory loss characteristic of AD. Current projections indicate that the incidence of dementia will double in Europe and triple globally by 2050. This alarming trend underscores the urgent need for effective preventive and therapeutic strategies.

AD is closely associated with inflammation, manifesting in the form of elderly plaques and activated microglia surrounding these plaques (Cuicui et al. 2023; Yetirajam and Thirumala‐Devi 2022). This inflammatory response is a critical component of the disease pathology, contributing to the progressive neurodegeneration and cognitive decline characteristic of AD. Evidence suggests that the pathological changes in neuroinflammation and tau protein are intricately linked to cognitive dysfunction, exhibiting complex and close interactions. The sustained and escalating inflammatory response of glial cells and neurons serves as a critical cellular driver and regulator of tau pathological deterioration, which, in turn, exacerbates tau pathology by intensifying the inflammatory response (Yijun and Yang 2023).

The role of inflammation in AD has been further supported by epidemiological studies, which have demonstrated that anti‐inflammatory drugs have the potential to reduce the incidence rate of AD (Sujata et al. 2022). This has spurred significant interest in developing anti‐inflammatory therapies as a potential treatment strategy. Promising drug therapies are currently in the late stages of clinical trials, encompassing strategies targeting amyloid beta, tau, and inflammation (Philip et al. 2021). For instance, peroxisome proliferator‐activated receptor γ (PPARγ) agonists like pioglitazone enhance the phagocytosis of Aβ and decrease the inflammatory cytokine IL‐1β (Interleukin‐1β). These drugs modulate the immune response and reduce the levels of pro‐inflammatory molecules, thereby potentially slowing disease progression. Additionally, several drugs, including feglastin, epigallocatechin gallate, curcumin, nicergoline, and minocycline, are under active development (Sneha et al. 2023). These compounds have shown promise in preclinical studies for their anti‐inflammatory and neuroprotective properties. However, the clinical trials of anti‐inflammatory drugs have not yielded uniformly successful outcomes (Tomris and Serkan 2019). Despite the potential benefits observed in preclinical models and epidemiological studies, translating these findings into effective treatments has proven challenging. The variability in patient responses, the complexity of the disease, and the potential for off‐target effects have all contributed to the difficulties in achieving consistent therapeutic success. Given this, it is imperative to identify more druggable targets and develop therapeutic drugs with greater promise.

**FIGURE 1:**
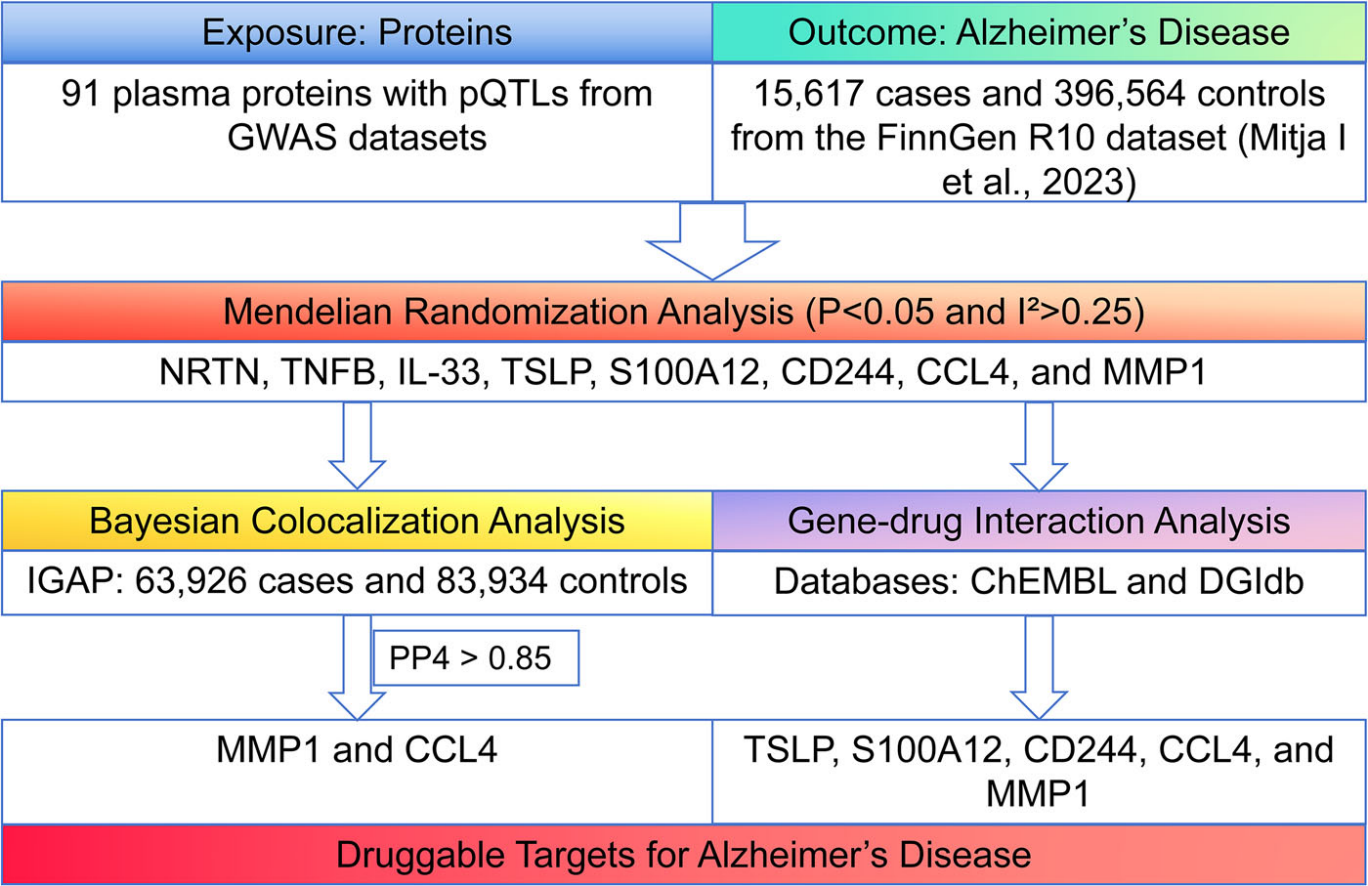
Study design for identification of druggable targets causally associated with AD. **Abbreviations**: CCL4, C‐C motif chemokine 4; CD244, Natural killer cell receptor 2B4; GWAS, Genome‐Wide Association Study; IGAP, International Genomics of Alzheimer's Project; IL33, Interleukin‐33; MMP1, Matrix metalloproteinase‐1; NRTN, Neurturin; pQTLs, protein quantitative trait loci; S100A12, Protein S100‐A12; TNFB, Tumor necrosis factor β; TSLP, Thymic stromal lymphopoietin. Heterogeneity was evaluated using Cochran *Q*‐test and the *I* ^2^ statistics, where a *p*‐value less than 0.05 and an *I* ^2^ greater than 0.25 were considered significant.

Mendelian randomization (MR) leverages genetic variation as instrumental variables to establish robust causal relationships between exposures and clinical phenotypes. Given its capacity for inferring causality, MR is increasingly employed to identify potential therapeutic targets by pinpointing genes with causal effects on disease outcomes, thereby providing a theoretical basis and direction for novel drug development (Kang‐Fu et al. 2024; Naiqi et al. 2024; Wei‐Ming et al. 2023). Although MR is extensively employed for genetic causal inference in Alzheimer's disease, its utility for therapeutic target identification is limited by susceptibility to weak instrument bias, unresolved horizontal pleiotropy, and failure to interrogate tissue‐specific mechanisms. Critically, while MR establishes genetic‐level associations, it neither confirms shared causal variants between exposure and outcome nor addresses key drug development dimensions. To overcome these constraints, we integrated Bayesian colocalization with druggability screening using ChEMBL (Qian et al. 2025; Shangyun et al. 2025) and DGIdb databases, thereby validating MR‐derived causal genes and prioritizing targets with mechanistic and therapeutic relevance. The methodological framework is schematically outlined in Figure 1.

## 2 Methods

### 2.1 Mendelian Randomization Analysis

The GWAS datasets were obtained for 91 plasma proteins, involving 14,824 healthy subjects of European ancestry. This comprehensive GWAS identified 180 protein quantitative trait loci (pQTLs) that significantly influenced circulating cytokine levels. For the MR analysis, corresponding GWAS data for each of the 91 proteins examined in this study were utilized, encompassing whole genome single nucleotide polymorphisms (SNPs) from 12,824 subjects. The summary results for AD included 15,617 cases and 396,564 controls from the FinnGen R10 dataset (Mitja et al. 2023).

Causal effects were determined using three models from the TwoSampleMR package (version 0.5.6): the inverse variance weighted (IVW) model, the weighted median (WM) model, and the MR Egger model. These models were employed as additional measures to ensure sensitivity and robustness of the findings. The variability was assessed using the intercept of MR Egger regression. A significant deviation between the MR Egger intercept and zero indicated that certain instrumental variables (IVs) were invalid and potentially biased the results. Heterogeneity was evaluated using the Cochran *Q*‐test and *I* ^2^ statistics, with thresholds set at *p* < 0.05 and *I* ^2^ > 0.25, respectively.

In our study, we utilized 91 plasma proteins as exposures and AD as the outcome. For each exposed phenotype, SNPs significantly associated with the exposure (*p* < 1×10^−5^) were selected. These SNPs were then pruned within a 10 Mb window, ensuring a clustered *r* ^2^ value of 0.001, and subsequently used as instrumental variables (IVs). This rigorous approach aimed to minimize the risk of confounding and ensure the validity of the causal inferences drawn from the MR analysis.

### 2.2 Bayesian Colocalization Analysis

Bayesian colocalization analysis was performed using Alzheimer's disease GWAS summary statistics from the International Genomics of Alzheimer's Project (IGAP; 63,926 cases and 83,934 controls) and multi‐tissue expression quantitative trait loci (eQTL) data from GTEx version 8 (prefrontal cortex, hippocampus, whole blood, and monocytes). For each target from MR analysis, we analyzed genomic regions spanning ± 500 kb from the transcription start site. Posterior probabilities for five mutually exclusive hypotheses were computed using the coloc.abf algorithm (R package coloc v5.1.0): (1) H0: No association with either trait; (2) H1: Association with AD only; (3) H2: Association with eQTL only; (4) H3: Two independent causal associations; (5) H4: Shared causal variant. Where PP4 (PP.H4) quantified evidence for colocalization.

### 2.3 Gene‐Drug Interaction Analysis

In our comprehensive effort to identify proteins with causal effects on AD, we conducted meticulous searches within the drug‐gene interaction databases ChEMBL (https://www.ebi.ac.uk/chembl/) and DGIdb (https://dgidb.org/) (Matthew et al. 2023). Our primary objective was to pinpoint specific inflammatory cytokines that are targeted by approved drugs, thereby identifying potential therapeutic avenues for AD. By leveraging these databases, we aimed to identify drugs that are already in clinical use or in advanced stages of development, which could be repurposed or further optimized for treating AD.

## 3 Results

### 3.1 MR Analysis

Our MR analysis revealed eight inflammatory proteins exhibiting statistically significant causal associations with AD. The IVs were extracted from exposure datasets as follows: 41 IVs for tumor necrosis factor‐β (TNFB), 25 IVs for Neurturin (NRTN), 24 IVs for Thymic stromal lymphopoietin (TSLP), 30 IVs for C‐C motif chemokine ligand 4 (CCL4C), 23 IVs for Calcium‐binding protein S100‐A12 (S100A12), 33 IVs for Natural killer cell receptor 2B4 (CD244), 23 IVs for IL33 (IL33), and 26 IVs for Matrix metalloproteinase‐1 (MMP1).

**FIGURE 2:**
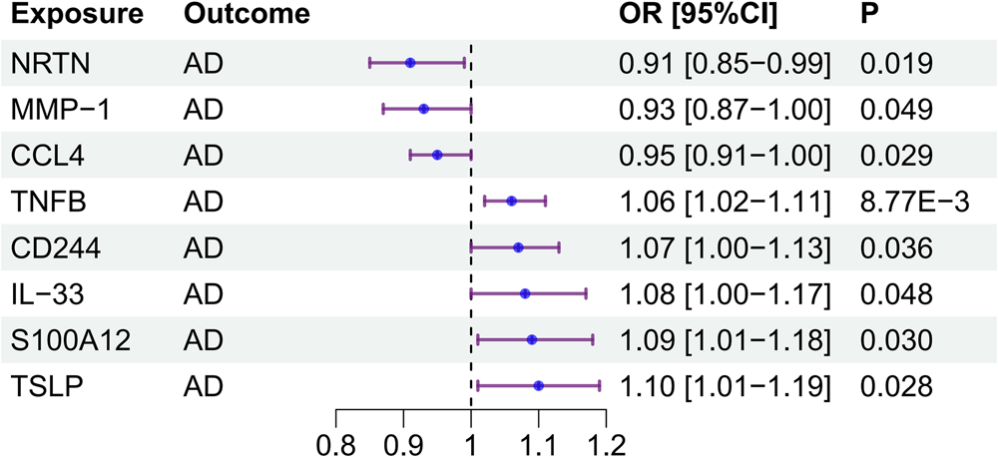
Effects of the core inflammatory proteins on the risk of AD. TSLP, S100A12, IL33, CD244, and TNFB were identified as proteins associated with elevated AD risk. Conversely, CCL4, MMP‐1, and NRTN proteins exhibited a protective effect against AD.

**FIGURE 3:**
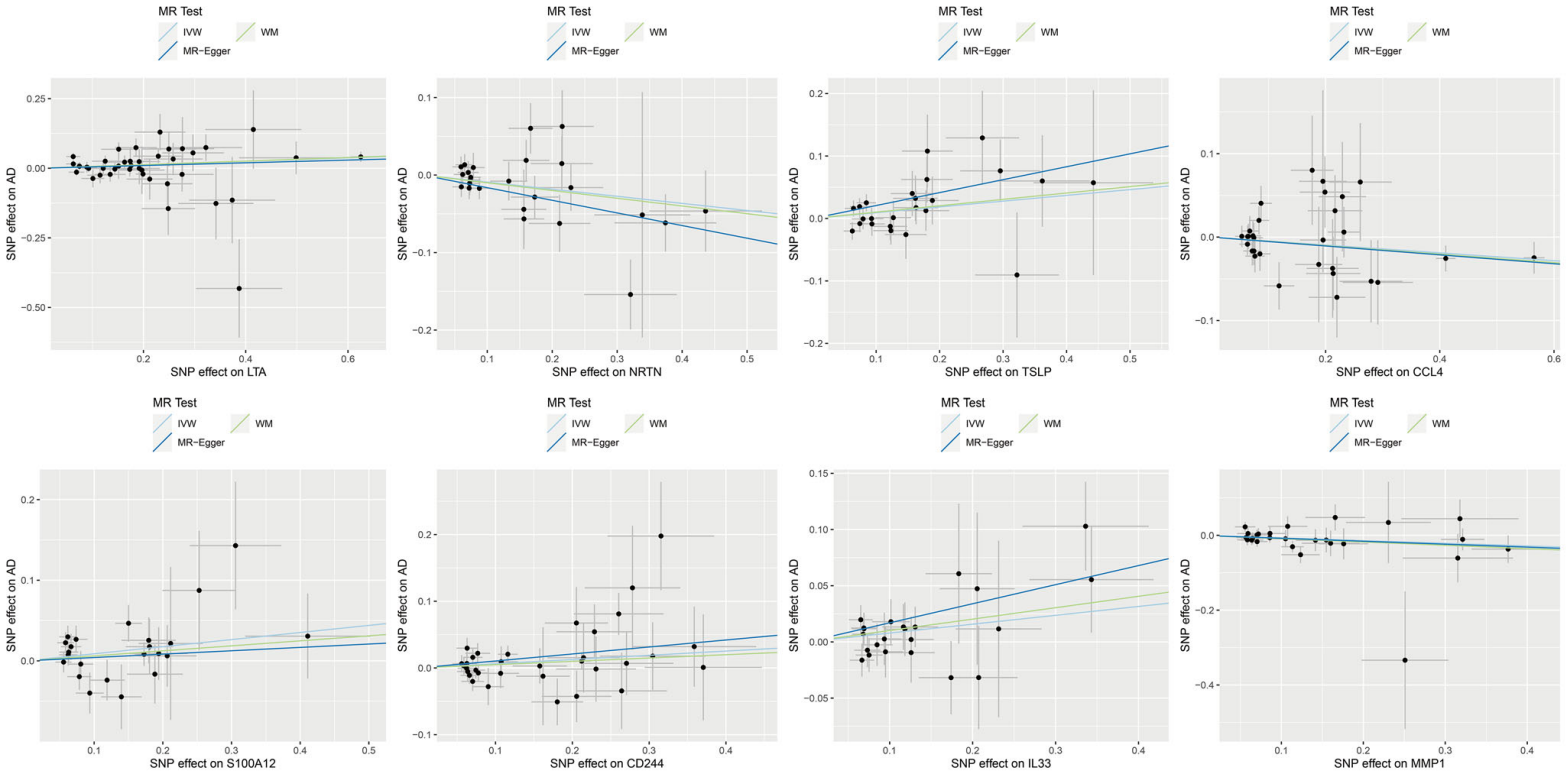
Scatter plots showing the causal relationships between circulating inflammatory proteins and AD using MR analysis. The plots are generated using two primary methods: IVW and WM.

More detailed results are presented in Table 1, Figure 2, and Figure 3.

**TABLE 1:** Significant results from the MR analysis in the discovery samples.

Exposure	Outcome	Method	N_IV	b(SE)	OR [95% CI]	*p*	Direction of effect
TNFB	AD	IVW	41	0.062 (0.024)	1.06 [1.02–1.11]	0.009	Increased risk
		WM	41	0.064 (0.028)	1.07 [1.01–1.13]	0.021	
		MR‐Egger	41	0.049 (0.037)	1.05 [0.98–1.13]	0.200	
NRTN	AD	IVW	25	−0.091 (0.039)	0.91 [0.85‐0.99]	0.019	Protective effect
		WM	25	−0.100(0.054)	0.91 [0.81‐1.01]	0.063	
		MR‐Egger	25	−0.163 (0.071)	0.85 [0.74‐0.98]	0.031	
TSLP	AD	IVW	24	0.092 (0.042)	1.10 [1.01‐1.19]	0.028	Increased risk
		WM	24	0.102 (0.061)	1.11 [0.98‐1.25]	0.094	
		MR‐Egger	24	0.207 (0.099)	1.23 [1.01‐1.49]	0.049	
CCL4	AD	IVW	30	−0.047 (0.022)	0.95 [0.91‐1.00]	0.029	Protective effect
		WM	30	−0.051 (0.028)	0.95 [0.90‐1.00]	0.067	
		MR‐Egger	30	−0.053 (0.030)	0.95 [0.89‐1.01]	0.089	
S100A12	AD	IVW	23	0.088 (0.041)	1.09 [1.01–1.18]	0.030	Increased risk
		WM	23	0.062 (0.056)	1.06 [0.95–1.19]	0.267	
		MR‐Egger	23	0.042 (0.083)	1.04 [0.89–1.23]	0.619	
CD244	AD	IVW	33	0.063 (0.030)	1.07 [1.00–1.13]	0.036	Increased risk
		WM	33	0.050 (0.050)	1.05 [0.95–1.16]	0.321	
		MR‐Egger	33	0.105 (0.059)	1.11 [0.99–1.25]	0.084	
IL33	AD	IVW	23	0.079 (0.040)	1.08 [1.00–1.17]	0.048	Increased risk
		WM	23	0.101 (0.058)	1.11 [0.99–1.24]	0.082	
		MR‐Egger	23	0.170 (0.092)	1.19 [0.99–1.42]	0.078	
MMP1	AD	IVW	26	−0.071 (0.036)	0.93 [0.87‐1.00]	0.049	Protective effect
		WM	26	−0.089 (0.052)	0.91 [0.83‐1.01]	0.085	
		MR‐Egger	26	−0.079 (0.068)	0.92 [0.81‐1.06]	0.255	

**Abbreviations**: b (SE), b (β)‌; CI, confidence interval; IVW, inverse variance weighted; OR, odds ratio; N_IV: number of instrumental variables;, Measure of the genetic association between a SNP and the exposure/outcome variable. SE, standard error;‌: WM, weighted median. Estimate of the variability in the beta value, used to calculate statistical significance and confidence intervals.

Table 1 provides a concise summary of the significant results from the MR analysis, highlighting the causal relationships between specific circulating inflammatory proteins and AD risk. The findings underscore the importance of targeting these proteins in the development of therapeutic strategies for AD, with both risk‐increasing and protective factors identified. Increased Risk: Proteins such as TSLP, S100A12, IL33, CD244, and TNFB are associated with an increased risk of AD, as indicated by their odds ratios (OR) greater than 1 and statistically significant *p*‐values. Protective Effect: Proteins such as CCL4, MMP‐1, and NRTN exhibit a protective effect against AD, as indicated by their OR less than 1 and statistically significant P‐values.

The forest plot in Figure 2 highlights the differential effects of core inflammatory proteins on AD risk. Proteins such as TSLP, S100A12, IL33, CD244, and TNFB are associated with an elevated risk of AD, suggesting that targeting these proteins may offer potential therapeutic strategies for mitigating AD progression. Conversely, CCL4, MMP‐1, and NRTN exhibit protective effects, indicating that these proteins may play a role in reducing AD risk and could be explored further as potential therapeutic targets.

Figure 3 provides a visual representation of the causal relationships between circulating inflammatory proteins and AD risk, as estimated by the IVW and WM methods. The scatter plots highlight the differential effects of various proteins, with some exhibiting a positive causal relationship (elevated AD risk) and others showing a negative causal relationship (protective effect against AD). These findings underscore the importance of targeting specific inflammatory pathways in the development of novel therapeutic strategies for AD.

### 3.2 Bayesian Colocalization Analyses

Strong colocalization evidence (PP4 > 0.85) was observed for MMP1 (PP4 = 0.92) and CCL4 (PP4 = 0.87) in the prefrontal cortex. CD244 (PP4 = 0.28) and IL‐33 (PP4 = 0.31) showed moderate evidence in whole blood, while NRTN, TNFB, TSLP, and S100A12 exhibited no significant colocalization (PP4 ≤ 0.23). The lead variant rs1799750 (MMP1) had SNP.PP.H4 = 0.95. As shown in Table 2.

**TABLE 2:** Bayesian colocalization results for AD‐associated proteins.

No.	Gene	Tissue	PP4	Top variant (SNP.PP.H4)	AD association
1	NRTN	Prefrontal ctx	0.18	rs8102766 (0.22)	Protective
2	TNFB	Whole blood	0.10	rs909253 (0.15)	Risk
3	IL‐33	Hippocampus	0.31	rs11740584 (0.40)	Risk
4	TSLP	Prefrontal ctx	0.23	rs3806933 (0.31)	Risk
5	S100A12	Prefrontal ctx	0.22	rs1750547 (0.29)	Risk
6	CD244	Whole blood	0.28	rs3766379 (0.36)	Risk
7	CCL4	Prefrontal ctx	0.87	rs1719217 (0.91)	Protective
8	MMP1	Prefrontal ctx	0.92	rs1799750 (0.95)	Protective

### 3.3 Gene‐Drug Interaction Analysis

The results of the gene‐drug interaction analysis conducted on the key proteins identified in the MR analysis are presented in Table 3. The analysis was performed by searching the drug‐gene interaction databases ChEMBL and DGIdb. Among prioritized proteins, TSLP, S100A12, CD244, CCL4, and MMP1 demonstrated druggability. The findings suggest that targeting these proteins could offer promising therapeutic strategies for managing and potentially preventing AD.

**TABLE 3:** Gene‐Drug interaction analysis for key proteins identified in the MR analysis.

Gene	Protein name	Drug	Max phase for indication	Regulatory approval	Indications	Interaction score
TSLP	Thymic stromal lymphopoietin	Tezepelumab	4	Approved	Asthma, airway obstruction	52.50
		AMG 157	2		Asthma	26.25
S100A12	Protein S100‐A12	Atogepant	4	Approved	Migraine disorder	5.25
		Methotrexate	4	Approved	Childhood acute lymphoblastic leukemia/acute lymphoblastic leukemia/lymphoblastic lymphoma, polyarticular arthritis/arthritis, cancer/neoplasm, psoriasis/psoriasis vulgaris, psoriatic arthritis, rheumatoid arthritis, breast cancer/breast neoplasm/breast carcinoma, choriocarcinoma, juvenile idiopathic arthritis, lung cancer, immune system disease, non‐Hodgkin's lymphoma, mycosis fungoides, leptomeningeal metastasis, fungal infectious disease, adenoma	0.18
		Ubrogepant	4	Approved	Migraine disorder	5.25
		Rimegepant	4	Approved	Migraine disorder	3.50
		Eptinezumab	4	Approved	Migraine disorder	3.50
CD244	Natural killer cell receptor 2B4	Aldesleukin	4	Approved	Cancer/neoplasm, metastatic melanoma/cutaneous melanoma/melanoma	3.50
IL‐33	Interleukin‐33	Itepekimab	3		Chronic obstructive pulmonary disease	13.12
			2		Atopic eczema, asthma	
		Torudokimab	2		Atopic eczema	13.12
		Etokimab	2		Sinusitis, peanut allergic reaction, atopic eczema, asthma	13.12
			1		Inflammatory abnormality of the skin	
		Astegolimab	3		Chronic obstructive pulmonary disease	4.37
CCL4	C‐C motif chemokine 4	Clodronic acid	4	Approved	Antineoplastic agent	3.18
		Cyclosporine	4	Approved	Immunosuppressant, ophthalmological agent	0.40
		Epoetin alfa	4	Approved	For treatment of anemia, antianemic agents, for treatment of stroke, erythropoietic agents	1.02
MMP1	Matrix metalloproteinase‐1	Ribavirin	4	Approved	Chronic hepatitis C virus infection, cirrhosis of the liver/fibrosis, cirrhosis of the liver, viral disease	0.13
		Interferon beta	4	Approved	Antiasthmatic agent for treatment of multiple sclerosis	1.31
		Doxycycline anhydrous	4	Approved	Gonorrhea, typhus, tularemia, brucellosis, periodontitis	0.11
		Hydrocortisone butyrate	4	Approved	Seborrheic dermatitis, atopic eczema, skin disease	0.12
		Leuprolide acetate	4	Approved	For treatment of Alzheimer's disease, for treatment of endometriosis, antineoplastic agent	0.52
		Triamcinolone	4	Approved	For treatment of diabetic macular edema	0.22
		Leflunomide	4	Approved	Antirheumatic Agents	0.43

## 4 Discussion

Neuroinflammation has gained significant recognition as a critical driver in the onset and progression of neurodegenerative disorders, with AD being a prominent example (Wenwen et al. 2024). Recent advances in genomics and proteomics have provided new insights into the role of inflammation in AD pathogenesis, highlighting a genetically anchored causal nexus between levels of circulating inflammatory cytokines and the risk of neurodegenerative diseases. This study employs MR to investigate the causal relationships between 91 circulating inflammatory proteins and AD, identifying several inflammatory proteins that significantly contribute to AD risk. Specifically, TNFB, TSLP, S100A12, CD244, and IL33 were associated with an increased risk of AD, while NRTN, CCL4, and MMP1 exhibited protective effects.

TSLP is closely linked to AD, with its interaction with vascular cell adhesion molecule‐1 (VCAM‐1) highlighting its role in AD progression (Jian et al. 2023; Tornike and Jonathan 2023). In this study, TSLP emerged as a potential therapeutic target, supported by its high interaction score. S100A12 contributes to inflammation and protein aggregation in AD, with its association with immune microenvironment changes further emphasizing its importance (Lai et al. 2022; Shepherd et al. 2006). Atomoxetine has shown promise in modulating inflammation‐related factors like CD44, potentially halting AD progression (Allan et al. 2021). IL‐33 has demonstrated neuroprotective effects in AD models by enhancing microglia‐mediated Aβ clearance and reducing pro‐inflammatory gene expression (Amy K Y et al. 2016; Taotao et al. 2023; Zhi et al. 2014). NRTN, delivered via AAV2 vectors, promotes dopamine neuron survival and resistance to toxicity, offering potential for AD treatment (Christopher et al. 2011; Christopher D et al. 2013). Meanwhile, CCL4 plays a role in amyloid processing and inflammation, with Andrographolide showing efficacy in reducing its expression (John S. K. et al. 2014; V. Julie et al. 2017; Zhang et al. 2021). Matrix metalloproteinases (MMPs), particularly MMP1, are linked to AD, with elevated levels in AD patients suggesting their role as biomarkers (Erik et al. 2021; H. Julie et al. 2021). MMP‐1 levels correlate with oxidative stress and cognitive decline, and Scutellaria baicalensis Georgi has shown potential in delaying AD progression by reducing MMP‐1 secretion (Li et al. 2022; Sylwia et al. 2022). Repetitive transcranial magnetic stimulation (rTMS) targeting the dorsolateral prefrontal cortex may regulate the MMPs/TIMPs system, delaying AD progression in mild cognitive impairment (MCI) patients (Giovanni et al. 2023). Bayesian colocalization analysis demonstrated strong genetic evidence (PP4 > 0.85) for shared causal mechanisms between MMP1 and CCL4 with AD protection in the prefrontal cortex, establishing them as priority therapeutic targets and further verifying the results of MR analysis.

After extensively exploring the drug‐gene interaction databases ChEMBL and DGIdb, we uncovered some promising findings. Among the key proteins we investigated, TSLP, S100A12, CD244, CCL4, and MMP1 emerged as druggable proteins. This breakthrough suggests that these proteins could potentially be targeted for the treatment of AD.

Cyclosporin ameliorates CCL4‐mediated neuroinflammation by inhibiting the calcineurin/NFAT signaling pathway (Joanna et al. 2023), which concurrently restores mitochondrial membrane potential dynamics and fission‐fusion balance (Wenqiang et al. 2024). This dual mechanism attenuates β‐amyloid neurotoxicity, protects neurons from apoptosis, and ultimately rescues AD‐related cognitive impairment, positioning cyclosporin as a promising therapeutic candidate warranting further clinical evaluation for AD.

Leuprolide acetate, a powerful drug targeting MMP1, has demonstrated remarkable potential in inhibiting pituitary gonadotropin secretion and suppressing the production of testicular or ovarian steroids over extended periods. It has proven effective in treating a range of sex hormone‐dependent conditions, including prostate cancer, endometriosis, uterine fibroids, and precocious puberty. Additionally, it has shown promise in improving symptoms in patients with AD. Notably, clinicians have observed cognitive improvements in patients with AD and prostate cancer while using leuprolide (Tracy et al. 2021). These compelling findings strongly support the potential of using MMP1 as a target for AD treatment.

These findings underscore the therapeutic potential of targeting specific inflammatory pathways in Alzheimer's disease, particularly through genetically validated mechanisms. Our integrated analysis identifies druggable targets, especially MMP1 and CCL4, that offer tractable avenues for immune‐modulating therapies. This approach advances the development of mechanistically informed treatments with significant potential to modify AD progression.

This study has several noteworthy constraints: reliance on Eurocentric GWAS data restricts ancestral generalizability (particularly regarding East Asian populations); unresolved methodological biases in MR analysis, including residual horizontal pleiotropy and non‐negligible weak instrument bias for proteins with limited cis‐pQTLs, persist despite sensitivity testing; and conventional druggability screening (ChEMBL/DGIdb) may overlook emerging therapeutic paradigms like RNA‐targeting approaches applicable to traditionally undruggable proteins. Consequently, while our integrative approach nominates promising targets, their translational relevance requires validation through mechanistic studies across diverse populations and next‐generation therapeutic platforms.

## 5 Conclusion

In our study, integrative genetic evidence suggests potential influences of circulating molecules on AD susceptibility. These proteins emerge as viable therapeutic targets, with MMP1 and CCL4 demonstrating prioritized candidacy based on strong colocalization evidence. This supports the development of immune‐focused therapeutic strategies, warranting further mechanistic exploration and clinical validation to advance targeted interventions for AD management.

## Author Contributions

**Hongliang An**: writing – original draft, writing – review and editing, visualization, and data curation. **Jianhong Gu**: methodology, visualization, and supervision. **Taiping Li**: project administration, software, resources, writing – review and editing.

## Ethics Statement

The authors have nothing to report.

## Conflicts of Interest

The authors declare no conflicts of interest.

## Peer Review

The peer review history for this article is available at https://publons.com/publon/10.1002/brb3.70797.

## Supporting information




**Supporting Table**: brb370797‐sup‐0001‐SuppMat.xlsx

## Data Availability

The original contributions presented in the study are included in the article/supporting information; further inquiries can be directed to the corresponding author.

## References

[brb370797-bib-0001] Agudelo‐Botero, M. , L. Giraldo‐Rodríguez , and M. E. Rojas‐Russell . 2023. “Systematic and Comparative Analysis of the Burden of Alzheimer´s Disease and Other Dementias in Mexico. Results at the National and Subnational Levels, 1990–2019.” The Journal of Prevention of Alzheimer's Disease 10, no. 1: 120–129. 10.14283/jpad.2022.92 36641616

[brb370797-bib-0002] Allan, I. , L. Deqiang , Q. Liping , et al. 2021. “A Phase II Study Repurposing Atomoxetine for Neuroprotection in Mild Cognitive Impairment.” Brain 145, no. 6: 1924–1938. 10.1093/brain/awab452 PMC9630662 34919634

[brb370797-bib-0003] Amy, K. Y. , K. W. Hung , M. Y. Yuen , et al. 2016. “IL‐33 Ameliorates Alzheimer's Disease‐Like Pathology and Cognitive Decline.” PNAS 113, no. 19: E2705–E2713. 27091974 10.1073/pnas.1604032113 PMC4868478

[brb370797-bib-0004] Christopher, D. , K. M. Bishop , L. Brown , et al. 2011. “Gene Transfer Provides a Practical Means for Safe, Long‐term, Targeted Delivery of Biologically Active Neurotrophic Factor Proteins for Neurodegenerative Diseases.” Drug Delivery and Translational Research 1, no. 5: 361–382. 25788422 10.1007/s13346-011-0037-z

[brb370797-bib-0005] Christopher, D. , L. Brown , B. R. Kruegel , et al. 2013. “Enhanced Neurotrophic Distribution, Cell Signaling and Neuroprotection Following Substantia Nigral versus Striatal Delivery of AAV2‐NRTN (CERE‐120).” Neurobiology of Disease 58, no. 0: 38–48. 23631873 10.1016/j.nbd.2013.04.011

[brb370797-bib-0006] Cuicui, W. , Z. Shuai , C. Xiaolin , et al. 2023. “The Effects of Microglia‐associated Neuroinflammation on Alzheimer's Disease.” Frontiers in Immunology 14, no. 0: 1117172. 36911732 10.3389/fimmu.2023.1117172 PMC9992739

[brb370797-bib-0007] Erik, B. , J. C. Adair , J. E. Knoefel , et al. 2021. “Inflammatory Biomarkers Aid in Diagnosis of Dementia.” Frontiers in Aging Neuroscience 13, no. 0: 717344. 34489684 10.3389/fnagi.2021.717344 PMC8416621

[brb370797-bib-0008] Giovanni, C. , P. Roberta , S. Mattia , et al. 2023. “Long‐Term Neuromodulatory Effects of Repetitive Transcranial Magnetic Stimulation (rTMS) on Plasmatic Matrix Metalloproteinases (MMPs) Levels and Visuospatial Abilities in Mild Cognitive Impairment (MCI).” International Journal of Molecular Sciences 24, no. 4: 3231. 36834642 10.3390/ijms24043231 PMC9961904

[brb370797-bib-0009] Jason, W. , and B. Andrew . 2018. “Current Understanding of Alzheimer's Disease Diagnosis and Treatment.” F1000Research 7, no. 0: F1000. 10.12688/f1000research.14506.1 PMC6073093 30135715

[brb370797-bib-0010] Jian, C. , D. An‐Xiang , T. Hai‐Liang , et al. 2023. “Increase of ALCAM and VCAM‐1 in the Plasma Predicts the Alzheimer's Disease.” Frontiers in Immunology 13, no. 0: 1097409. 36685605 10.3389/fimmu.2022.1097409 PMC9846483

[brb370797-bib-0011] Joanna, M. , L. Malwina , and B. Tomasz . 2023. “Targeting CaN/NFAT in Alzheimer's Brain Degeneration.” Frontiers in Immunology 14, no. 0: 1281882. 38077352 10.3389/fimmu.2023.1281882 PMC10701682

[brb370797-bib-0012] John, S. K. , M. H. Bailey , P. G. Ridge , et al. 2014. “Genome‐wide Association Study of CSF Levels of 59 Alzheimer's Disease Candidate Proteins: Significant Associations With Proteins Involved in Amyloid Processing and Inflammation.” PLos Genetics 10, no. 10: e1004758. 25340798 10.1371/journal.pgen.1004758 PMC4207667

[brb370797-bib-0013] Julie, H. , H. Elisabeth , E. Sebastiaan , et al. 2021. “Investigation of the Role of Matrix Metalloproteinases in the Genetic Etiology of Alzheimer's Disease.” Neurobiology of Aging 104, no. 0: 105.e1–105.e6. 10.1016/j.neurobiolaging.2021.03.011 33892965

[brb370797-bib-0014] Julie, V. , J. Thierry , J. Adrien , C. Damien , P. Guylene , and P. Marc . 2017. “Peripheral Blood Mononuclear Cells of Alzheimer's Disease Patients Control CCL4 and CXCL10 Levels in a Human Blood Brain Barrier Model.” Current Alzheimer Research 14, no. 11: 1215–1228. 28413983 10.2174/1567205014666170417110337

[brb370797-bib-0015] Kang‐Fu, Y. , C. Ting , G. Xiao‐Jing , et al. 2024. “Systematic Druggable Genome‐wide Mendelian Randomization Identifies Therapeutic Targets for Sarcopenia.” Journal of Cachexia, Sarcopenia and Muscle 15, no. 4: 1324–1334. 38644354 10.1002/jcsm.13479 PMC11294052

[brb370797-bib-0016] Kiara, F. , S. Nele , W. Sarah , et al. 2022. “Spermidine Reduces Neuroinflammation and Soluble Amyloid Beta in an Alzheimer's Disease Mouse Model.” Journal of Neuroinflammation 19, no. 1: 172. 35780157 10.1186/s12974-022-02534-7 PMC9250727

[brb370797-bib-0017] Lai, Y. , P. Lin , F. Lin , et al. 2022. “Identification of Immune Microenvironment Subtypes and Signature Genes for Alzheimer's Disease Diagnosis and Risk Prediction Based on Explainable Machine Learning.” Frontiers in Immunology 13: 1046410. 36569892 10.3389/fimmu.2022.1046410 PMC9773397

[brb370797-bib-0018] Lanctôt, K. L. , J. Hviid Hahn‐Pedersen , C. S. Eichinger , et al. 2024. “Burden of Illness in People With Alzheimer's Disease: A Systematic Review of Epidemiology, Comorbidities and Mortality.” The Journal of Prevention of Alzheimer's Disease 11, no. 1: 97–107. 10.14283/jpad.2023.61 PMC10225771 38230722

[brb370797-bib-0019] Li, G. , Y. Wu‐Yan , Q. Hong , S. Chang‐Jun , Q. Xue‐Mei , and D. Guan‐Hua . 2022. “Unveiling the Anti‐senescence Effects and Senescence‐associated Secretory Phenotype (SASP) Inhibitory Mechanisms of Scutellaria baicalensis Georgi in Low Glucose‐induced Astrocytes Based on Boolean Network.” Phytomedicine 99, no. 0: 153990. 35202958 10.1016/j.phymed.2022.153990

[brb370797-bib-0020] Matthew, C. , S. James , S. Kathryn , et al. 2023. “DGIdb 5.0: Rebuilding the Drug‐gene Interaction Database for Precision Medicine and Drug Discovery Platforms.” Nucleic Acids Research 52, no. D1: D1227–D1235. 10.1093/nar/gkad1040 PMC10767982 37953380

[brb370797-bib-0021] Mitja, I. , J. Karjalainen , P. Palta , et al. 2023. “FinnGen Provides Genetic Insights From a Well‐phenotyped Isolated Population.” Nature 613, no. 7944: 508–518. 36653562 10.1038/s41586-022-05473-8 PMC9849126

[brb370797-bib-0022] Naiqi, Z. , L. Yanni , S. Jan , S. Kristina , and J. Jianguang . 2024. “Identifying Actionable Druggable Targets for Breast Cancer: Mendelian Randomization and Population‐based Analyses.” EBioMedicine 98, no. 0: 104859. 10.1016/j.ebiom.2023.104859 PMC10628347 38251461

[brb370797-bib-0023] Philip, S. , D. S. Bart , K. Miia , et al. 2021. “Alzheimer's Disease.” Lancet 397, no. 10284: 1577–1590. 33667416 10.1016/S0140-6736(20)32205-4 PMC8354300

[brb370797-bib-0024] Qian, Z. , B. Ancha , L. Dongming , C. Hongbao , and Z. Fuquan . 2025. “Bidirectional Causal Associations Between Plasma Metabolites and Bipolar Disorder.” Molecular Psychiatry 30, no. 0: 3998–4005. 40169804 10.1038/s41380-025-02977-3

[brb370797-bib-0025] Shangyun, S. , B. Ancha , C. Hongbao , and Z. Fuquan . 2025. “Exploring Causal Associations Between Plasma Metabolites and Attention‐deficit/Hyperactivity Disorder.” BMC Psychiatry [Electronic Resource] 25, no. 1: 498. 40380147 10.1186/s12888-025-06951-9 PMC12084988

[brb370797-bib-0026] Shepherd, C. , J. Goyette , V. Utter , et al. 2006. “Inflammatory S100A9 and S100A12 Proteins in Alzheimer's Disease.” Neurobiology of Aging 27, no. 11: 1554–1563. 16253391 10.1016/j.neurobiolaging.2005.09.033

[brb370797-bib-0027] Sneha, K. , D. Rishika , and R. Dibbanti Harikrishna . 2023. “Apoptosis in Alzheimer's Disease: Insight Into the Signaling Pathways and Therapeutic Avenues.” Apoptosis 28, no. 0: 943–957. 37186274 10.1007/s10495-023-01848-y

[brb370797-bib-0028] Sujata, T. , D. Rishika , S. Phulen , M. Bikash , and R. Dibbanti Harikrishna . 2022. “Neuroinflammation in Alzheimer's Disease: Current Progress in Molecular Signaling and Therapeutics.” Inflammation 46, no. 1: 1–17. 35986874 10.1007/s10753-022-01721-1

[brb370797-bib-0029] Sylwia, B. , P.‐J. Anna , K. Katarzyna , S. Katarzyna , K. Jan , and G. Ewa . 2022. “The Concentration of Fibronectin and MMP‐1 in Patients With Alzheimer's Disease in Relation to the Selected Antioxidant Elements and Eating Habits.” Journal of Clinical Medicine 11, no. 21: 6360. 36362588 10.3390/jcm11216360 PMC9657660

[brb370797-bib-0030] Taotao, J. , Z. Ting , L. Wenhao , L. Ning , and W. Manxia . 2023. “IL‐33/ST2 Signaling Pathway and Alzheimer's Disease: A Systematic Review and Meta‐Analysis.” Clinical Neurology and Neurosurgery 230, no. 0: 107773. 37172376 10.1016/j.clineuro.2023.107773

[brb370797-bib-0031] Tomris, O. , and O. Serkan . 2019. “Neuro‐inflammation and Anti‐inflammatory Treatment Options for Alzheimer's Disease.” Clinical Biochemistry 72, no. 0: 87–89. 30954437 10.1016/j.clinbiochem.2019.04.001

[brb370797-bib-0032] Tornike, M. , and K. Jonathan . 2023. “Type 2 Immunity in the Brain and Brain Borders.” Cellular & Molecular Immunology 20, no. 11: 1290–1299. 37429945 10.1038/s41423-023-01043-8 PMC10616183

[brb370797-bib-0033] Tracy, B. , J. D. Goldberg , J. E. Galvin , et al. 2021. “Rationale, Study Design and Implementation of the LUCINDA Trial: Leuprolide plus Cholinesterase Inhibition to Reduce Neurologic Decline in Alzheimer's.” Contemporary Clinical Trials 107, no. 0: 106488. 34166841 10.1016/j.cct.2021.106488 PMC8550816

[brb370797-bib-0034] Wei‐Ming, S. , G. Xiao‐Jing , D. Meng , et al. 2023. “Systematic Druggable Genome‐wide Mendelian Randomisation Identifies Therapeutic Targets for Alzheimer's Disease.” Journal of Neurology, Neurosurgery, and Psychiatry 94, no. 11: 954–961. 37349091 10.1136/jnnp-2023-331142 PMC10579488

[brb370797-bib-0035] Wenqiang, Q. , L. Daozhou , L. Jie , et al. 2024. “The Mitochondria‐Targeted Micelle Inhibits Alzheimer's Disease Progression by Alleviating Neuronal Mitochondrial Dysfunction and Neuroinflammation.” Small 21, no. 6: e2408581. 39713820 10.1002/smll.202408581

[brb370797-bib-0036] Wenwen, L. , W. Xuewei , and O. Guanyong . 2024. “Causal Association of Circulating Inflammatory Proteins on Neurodegenerative Diseases: Insights From a Mendelian Randomization Study.” Journal of Cellular and Molecular Medicine 28, no. 20: e70176. 39470585 10.1111/jcmm.70176 PMC11520441

[brb370797-bib-0037] Yetirajam, R. , and K. Thirumala‐Devi . 2022. “Innate Immune Cell Death in Neuroinflammation and Alzheimer's Disease.” Cells 11, no. 12: 1885. 35741014 10.3390/cells11121885 PMC9221514

[brb370797-bib-0038] Yijun, C. , and Y. Yang . 2023. “Tau and Neuroinflammation in Alzheimer's Disease: Interplay Mechanisms and Clinical Translation.” Journal of Neuroinflammation 20, no. 1: 165. 37452321 10.1186/s12974-023-02853-3 PMC10349496

[brb370797-bib-0039] Zhang, J. , Y. Zheng , Y. Zhao , et al. 2021. “Andrographolide Ameliorates Neuroinflammation in APP/PS1 Transgenic Mice.” International Immunopharmacology 96: 107808. 34162168 10.1016/j.intimp.2021.107808

[brb370797-bib-0040] Zhao, Q. , A. Baranova , H. Cao , and F. Zhang . 2024. “Evaluating Causal Effects of Gut Microbiome on Alzheimer's Disease.” The Journal of Prevention of Alzheimer's Disease 11, no. 6: 1843–1848. 10.14283/jpad.2024.113 39559896

[brb370797-bib-0041] Zhi, X. , T. Ramasamy , K. Duraisamy , Y. Evert , Z. Smita , and Z. Asgar . 2014. “Alzheimer's Disease: Evidence for the Expression of Interleukin‐33 and Its Receptor ST2 in the Brain.” Journal of Alzheimer's Disease 40, no. 2: 297–308. 10.3233/JAD-132081 PMC4015800 24413615

